# Biomass Precursors for Hard Carbon Anodes in Sodium-Ion Batteries: Structural Characteristics and Performance Relationships

**DOI:** 10.3390/nano16140879

**Published:** 2026-07-16

**Authors:** Man Kang, Luyao Huang, Yuxuan Zhang, Fei Wang, Xiaowei Li, Xiaodong Wu

**Affiliations:** School of Materials Science and Engineering, Jiangsu University, Zhenjiang 212013, China; 2222405138@stmail.ujs.edu.cn (M.K.); 3240708131@stmail.ujs.edu.cn (L.H.); 3240708017@stmail.ujs.edu.cn (Y.Z.); wf6028@stmail.ujs.edu.cn (F.W.); lixw@ujs.edu.cn (X.L.)

**Keywords:** sodium-ion batteries, anode materials, biomass-derived carbon, structural regulation

## Abstract

Sodium-ion batteries (SIBs) have become a crucial supplementary technology for large-scale energy storage due to abundant sodium resources and their low cost. Biomass-derived hard carbon materials have been considered as one of the most promising anode materials for commercial SIBs. However, the inherent structures of different biomass materials vary significantly, which directly affects the electrochemical performance of the resulting hard carbon anode materials. In this review, raw materials are classified into four types based on the natural structure characteristics of biomass: fibrous, granular, dense, and special. This review highlights the structural characteristics of each biomass type and their influence on sodium storage performance. The carbonization process, including the treatment of raw materials before carbonization, the parameter control during the carbonization process, and the surface optimization after carbonization, is proposed as an effective strategy for regulating the structure of biomass-derived hard carbon. The existing structural deficiencies in current carbon materials are also analyzed. Finally, the selection of biomass precursors and the structural regulation strategies for commercial SIBs are discussed.

## 1. Introduction

The scarcity and uneven distribution of lithium resources have restricted the development of lithium-ion batteries (LIBs). Sodium-ion batteries (SIBs) have attracted much attention due to abundant sodium resources and their low cost [1,2]. The electrode material is a key component that affects the electrochemical performance of SIBs. The anode materials mainly include three categories: alloy materials, metal oxide materials, and carbon materials. Alloy anode materials offer high theoretical capacities but suffer from severe volume expansion during cycling. Metal oxide anode materials display high specific capacities and abundant elemental choices, but the poor electronic conductivity and large volume changes have limited their applications [3,4]. In contrast, carbon materials have the advantages of low cost, good conductivity, and adjustable structure [5,6]. Among them, hard carbon has become the most promising anode material due to its low redox potential and long cycle life [7,8,9,10]. However, hard carbon suffers from low coulombic efficiency and limited plateau capacity.

The precursors of hard carbon mainly include three types: biomass, synthetic polymers, and fossil fuels. Biomass material has a wide range of sources, including plants [8,11], agricultural waste [12,13,14,15,16,17], and algae, etc. The renewability of biomass materials and their inherent multi-level structure make them ideal precursors for the preparation of hard carbon. However, biomass also has limitations such as impurity interference, poor thermal stability, and an uneven structure. The natural structures of different biomass sources include fibrous, granular, and dense structures. These structural differences directly affect the final electrochemical performance of hard carbon anode material.

The current studies mainly focus on the electrochemical performance of different biomass materials. However, the systematic classification of structural features remains insufficient [18]. This work divides biomass into four categories, and then systematically summarizes the structural characteristics of each type of biomass on the electrochemical performance of hard carbon. The regulatory role of carbonization processes is also discussed. We hope that this review can provide a reference for the rational design of high-performance biomass carbon materials [19,20].

## 2. Sodium Storage Mechanism of Hard Carbon

The storage mechanism of hard carbon remains actively debated, particularly regarding the low-potential plateau region (<0.1 V). The current studies present multiple competing models: the classical adsorption–intercalation model, where Na^+^ sequentially adsorbs on defect sites and intercalates between graphite layers; the nanopore filling model, which attributes plateau capacity to quasi-metallic sodium cluster formation within closed pores; and hybrid interface-storage mechanisms that invoke coupled adsorption–intercalation–filling processes [21]. This diversity of perspectives reflects the complex and heterogeneous structure of hard carbon. Combining these models is expected to provide a more comprehensive overview [22].

**Figure 1:**
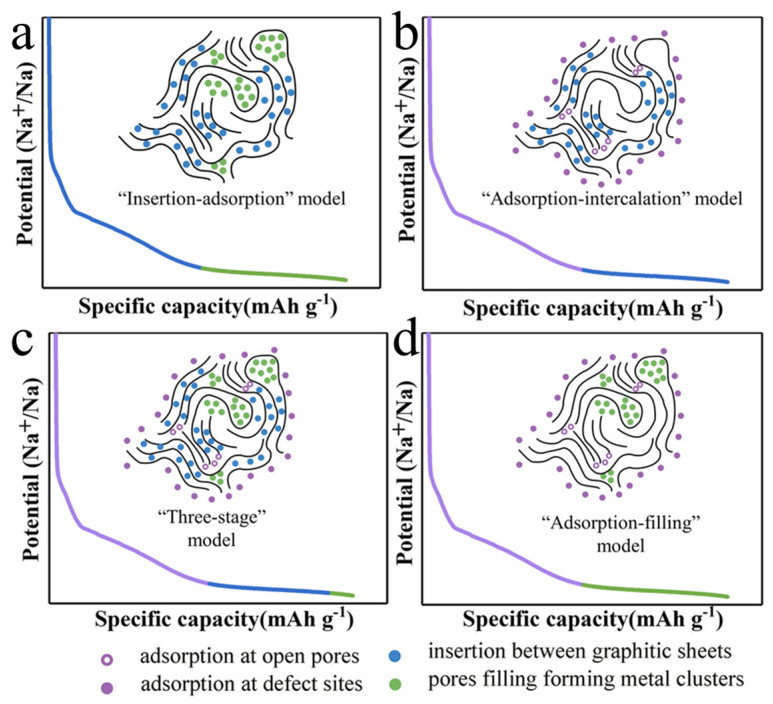
Summary of the proposed sodium storage models: (**a**) insertion-adsorption; (**b**) adsorption-intercalation; (**c**) three-stage/adsorption–intercalation-pore filling; (**d**) adsorption-pore filling Reprinted from Ref. [25].

The refined four-stage mechanism is shown in Figure 1a: (i) surface adsorption (>0.2 V); (ii) adsorption–intercalation (0.2–0.1 V); (iii) intercalation–filling (0.1–0.05 V); and (iv) intra-pore filling (<0.05 V). This staged change explains the abrupt drop in Na^+^ diffusivity at a moderate discharge depth [23]. The three stages jointly govern the storage of Na^+^. The interlayer spacing (d_002_) determines Na^+^ intercalation: values of 0.37–0.40 nm are optimal; below 0.36 nm intercalation is hindered; while above 0.40 nm the structure is too loose. Defects and specific surface area dominate the slope capacity and reduce initial coulombic efficiency (ICE), with a 5–10% ICE drop per 100 m g^−1^ increase in specific surface area. The closed pore volume is generally regarded as a key factor influencing the plateau capacity. However, the exact mechanisms, through quasi-metallic sodium cluster, micro-pore filling, or other pathways, remain unconfirmed. Notably, closed pores form in tandem with pseudo-graphitic-domain evolution during the high-temperature carbonization process. Moreover, pore entrance sizes < 0.35 nm exclude solvated Na^+^, confining solid electrolyte interphase (SEI) formation to the outer particle surface, preserving the internal closed pores [24,25]. Therefore, the electrochemical performance of hard carbon requires simultaneous multi-parameter optimization, such as d_002_, defect concentration, closed pore volume and pore entrance size. The above parameters mainly depend on the natural structure of the biomass precursor [26].

The key structural parameters of hard carbon are interlayer spacing, defects, and voids. The interlayer spacing is most favorable for Na^+^ transport between 0.37 and 0.40 nm [27,28]. Defects provide additional adsorption sites and mainly contribute to the slope capacity, but excessive defects also reduce the ICE of hard carbon. Void volume provides the capacity of the plateau. The relationship between these parameters and the sodium storage behavior is the basis for designing high-performance hard carbon. The natural structure of different biomass raw materials is a key factor influencing these parameters [29,30].

## 3. Diverse Morphology of Biomass-Derived Carbons for SIBs

**Figure 2:**
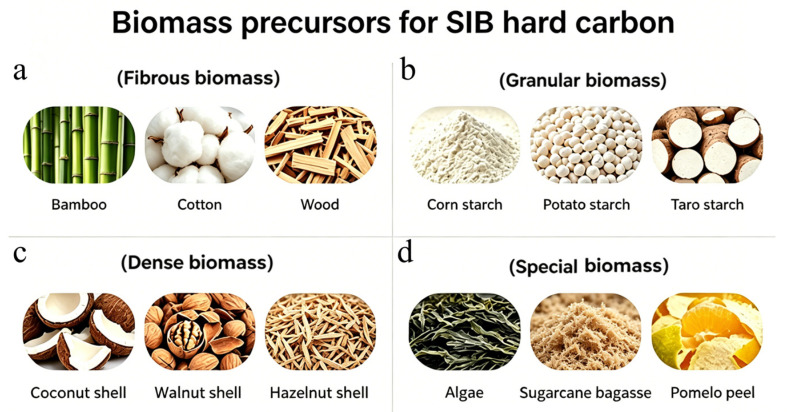
Classification of precursor materials for biomass-based hard carbon materials: (**a**) fibrous biomass; (**b**) granular biomass; (**c**) high-density biomass; (**d**) special biomass.

The natural structure of biomass exhibits multi-level ordered characteristics ranging from nanometers to micrometers [31]. As shown in Figure 2, the natural structures of different biomass will be partially retained after carbonization, forming unique hard carbon frameworks [32]. This section will discuss the natural structural types of biomass into four categories [33].

### 3.1. Fibrous Biomass

**Figure 3:**
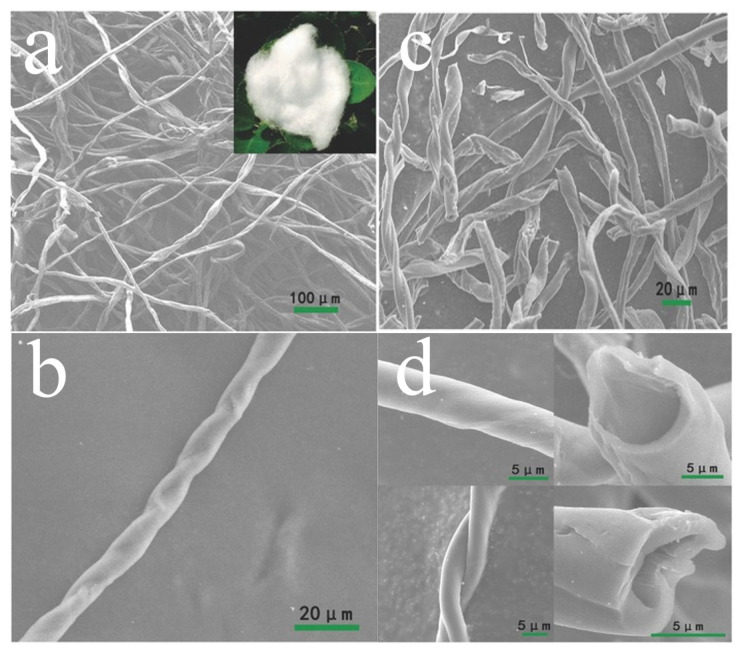
Morphology evolution of cotton before and after carbonization. (**a**) An SEM image of cotton (inset: a photograph of cotton); (**b**) a magnified SEM image of cotton; (**c**) an SEM image of the carbonized cotton; (**d**) magnified SEM images of the carbonized cotton with the detailed structural information. Reprinted with permission from Ref. [40]. Copyright [2016] [Yunming Li].

The fibrous structure is mainly found in the cell walls of higher plants, with cellulose being the representative [34,35,36]. Natural cellulose has a unique multi-level structure: primary fibrils of 1–3 nm aggregate to form microfibrils of 4–8 nm, which are then assembled into larger fibers, ultimately forming a three-dimensional network of the cell wall. In plant tissues, these cellulose fibers are further arranged into vascular bundle systems, forming macroscopic oriented channels [37]. After carbonization, the cellulose hard carbon can retain the fibrous or tubular morphology of the precursor. Xu et al. [38] found that the hard carbon derived from bamboo has a layer spacing of approximately 0.378 nm and a specific surface area of ~225 m^2^/g. Through the coordinated regulation of surface oxygen functional groups and closed micropores, an ICE of 86.1% and a reversible capacity of 326 mAh/g were achieved. Recent studies have further confirmed that the natural vascular bundle structure in bamboo can be partially retained after carbonization, forming straight-through pore channels. This bundle structure effectively shortens Na^+^ transport pathways and contributes to excellent rate capability [39]. As shown in Figure 3, Li et al. [40] prepare a uniform hollow microtube structure of hard carbon with 41% cotton fibers as the precursor. After carbonization at 1300 °C, the as-prepared sample exhibited the highest reversible capacity (315 mAh g^−1^) and 80–85% of charge–discharge efficiency, which was attributed to its unique tubular structure and an interlayer spacing of approximately 0.38 nm. Tang et al. [41] indicates that wood-derived hard carbon also retains the natural oriented pores. The main advantage of this fibrous raw material lies in the continuous fiber network, which provides rapid channels for electrons and ions. The optimized sample exhibited a reversible capacity of up to 430 mAh g^−1^ at 20 mA g^−1^, with high capacity retention of 85.4% after 400 cycles at 500 mA g^−1^. The above studies indicate that the retained natural structures, such as vascular bundles in bamboo, hollow microtubes in cotton, and closed pores in wood, play a crucial role in regulating the sodium storage performance of fiber biomass-derived hard carbon. However, natural plant fibers often contain impurities such as hemicellulose, lignin, and ash. These impurities may induce local graphitization or cause irreversible side reactions during the carbonization process. It is necessary to carry out appropriate pre-treatment to remove them [42,43].

### 3.2. Granular Biomass

**Figure 4:**
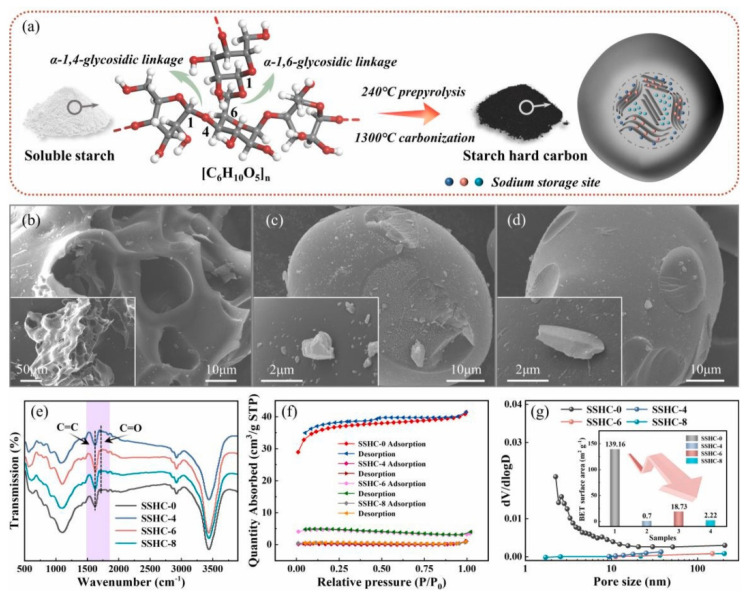
(**a**) Preparation process of soluble starch-derived hard carbon. SEM images of soluble starch-derived hard carbon, preheating for different durations: (**b**) 0 h; (**c**) 6 h; (**d**) 8 h. (**e**) FTIR spectra, (**f**) N_2_ adsorption-desorption isotherms, and (**g**) pore size distribution based on the BJH model of soluble starch-derived hard carbon with varying low-temperature pre-treatment durations Reprinted with permission from Ref. [48]. Copyright [2024] [Xiaotong Gao].

The granular structure is mainly found in starch-based biomass, including corn starch, potato starch, taro starch, etc. Starch is composed of amylopectin and amylose forming spherical or elliptical particles, which have a polyhydroxy structure and semi-crystalline properties [44,45]. This spherical particle structure with melting and foaming is prone to cause structural collapse during the direct high-temperature carbonization process. Therefore, direct carbonization of starch often results in amorphous carbon rather than hard carbon [46,47]. Appropriate pre-treatment can stabilize the spherical morphology of starch. Common pre-treatment methods include low-temperature pre-carbonization, enzymatic cross-linking, and yeast fermentation. The pre-treated starch particles can form isotropic spherical carbon particles with rich internal pore structures. Gao et al. [48] used potato starch as the raw material and adopted a low-temperature pre-treatment strategy to prepare hard carbon. The as-prepared sample has an appropriate spherical particle size, low specific surface area, and abundant internal pores, as shown in Figure 4. These favorable structural characteristics are conducive to high ICE and improved plateau capacity. The N_2_ adsorption–desorption isotherm of soluble starch-derived hard carbon obtained at different pre-treatment durations can be classified according to standards. The isotherms exhibit steep N_2_ uptake at very low relative pressure (P/P_0_ < 0.01), characteristic of Type I(b) behavior, indicating the presence of abundant micropores (<2 nm). The distinct hysteresis loop observed in the P/P_0_ range of approximately 0.4–0.9 is attributable to capillary condensation within mesopores (2–50 nm). The loop shape is typically associated with narrow slit-like pores or disordered micro-mesoporous structures with limited pore connectivity. At high relative pressure, the lack of a well-defined plateau suggests that some degree of pore constriction or ink-bottle pore geometry may be present in hard carbon [49].

The pore size distributions (Figure 4g) were calculated using the BJH method applied to the desorption branch. However, it is important to acknowledge that BJH analysis, which is based on the Kelvin equation, has well-known limitations for predominantly microporous materials. In the micropore (<2 nm) regime, capillary condensation does not follow the Kelvin equation, and BJH tends to systematically underestimate pore sizes and pore volumes [50]. For more accurate characterization of microporous hard carbons, the Density Functional Theory (DFT) model is recommended.

The pore connectivity, inferred from the combined isotherm and hysteresis analysis, has direct impact on sodium storage of hard carbon [51]. A well-connected pore network promotes electrolyte infiltration and Na^+^ transport. The poorly connected pores may be indistinguishable in N_2_ adsorption due to kinetic accessibility limitations, which will affect the analysis of the pore volume for Na^+^ storage. Notably, N_2_ adsorption only probes pores that are accessible to N_2_ molecules at 77 K. Closed pores that are not accessible to N_2_ may still be accessible to solvated Na^+^ ions in the electrolyte. This distinction underscores the need for complementary techniques such as small-angle X-ray scattering (SAXS) or CO_2_ adsorption to obtain a complete picture of the pore architecture.

Yang et al. [52] prepare porous spherical hard carbon via enzymatic hydrolysis combined with heat treatment with taro starch as the carbon source. The main advantage of these granular raw materials lies in their ease of forming pores. The isotropic structure of spherical particles is conducive to generating uniformly distributed pores during carbonization, thereby contributing to high plateau capacity. However, the thermal stability of granular material is poor, and appropriate pre-treatment is necessary to exert its structural advantages [53,54,55].

### 3.3. Dense Biomass

The dense structure is mainly found in shell-based biomass, including coconut shells, walnut shells, rice husks, hazelnut shells, etc. These raw materials have the characteristics of being dense, high in carbon content, and low in ash content. Unlike cellulose and starch, shell-based raw materials are highly ordered natural composite materials with abundant pore structures. In this composite structure, cellulose provides the framework, lignin offers rigidity and thermal stability, and hemicellulose serves as the filler. The synergistic effect of three components prevents shell-based raw materials from deforming during the carbonization process, thereby facilitating the formation of rich closed-pore structures [56,57]. Coconut shell is the most promising biomass hard carbon material for industrialization. Huang et al. [58] and Nita et al. [59] demonstrated that the proportion of lignin, cellulose and hemicellulose in coconut shells is balanced. The closed-cell structure formed after carbonization helps to increase the plateau capacity. Rybarczyk et al. [60] indicated that rice husk hard carbon exhibits excellent rate performance due to its natural layered porous structure. This dense raw material has the advantages of low ash content, high carbonization yield, and a well-developed closed-cell structure, thus being suitable for industrial applications [61,62]. However, the supply of coconut shell raw materials is the main bottleneck for large-scale application.

### 3.4. Special Types of Biomass

Special types of biomass mainly refer to emerging waste materials or invasive plants, including algae, coffee grounds, sugarcane residue, pomelo peels, and golden alexia [63,64]. These raw materials usually have inherent porous or layered structures, and compositional characteristics. For example, they naturally contain various non-metallic elements such as nitrogen and sulfur. The cell walls of algae are rich in proteins. Pomelo peels have a three-dimensional honeycomb structure, and sugarcane residue has a fiber network. After carbonization, these special structures retain the porous or layered carbon framework, while achieving in situ doping of N or S elements. Cha et al. [65] indicates that algae-derived hard carbon with natural nitrogen doping has abundant active sites and a high specific capacity. Rath et al. [66] prepared hard carbon with sugarcane bagasse as the precursor, which retained a fibrous network structure. Hong et al. [67] discovered that citrus peel-derived hard carbon has a three-dimensional connected porous structure, providing abundant storage sites for Na^+^. Wei et al. [68] prepared hard carbon with invasive plants as raw materials, such as salt marsh cordgrass and Canada thistle. The main advantages of these special types of raw materials are waste utilization, environmental friendliness, and in situ doping of elements. However, the extremely unstable source and significant performance fluctuations have limited its large-scale production [69].

### 3.5. From Natural Structure to Final Microstructure: Evolution of Key Parameters

Due to the different structures and compositions of the above four materials, the parameters in the preparation process vary significantly. The electrochemical performance is dictated by the interplay of natural precursor features (cellulose/lignin ratio, ash content, native porosity) and carbonization parameters. Conduct a comparative analysis of the structural evolution parameters during the carbonization process. The four biomass types differ fundamentally in how their carbonization products evolve.

(1) Interlayer spacing: Below 1000 °C, all precursors give d_002_ > 0.40 nm. Fibrous biomass (oriented cellulose) tends to induce preferential graphitization along the fiber axis, while granular starch yields isotropic spheres with slower d_002_ contraction. Dense, lignin-rich shells maintain rigidity and suppress over-graphitization, preserving d_002_ in the ideal 0.37–0.40 nm window even at 1400–1600 °C [70].
(2) Defects and specific surface area: Pre-oxidation (200–280 °C for lignocellulose, 150–250 °C for starch) introduces cross-linking oxygen groups that raise carbonization yield and produce a more ordered carbon framework [23]. Slow heating (0.5 °C min^−1^) allows defect annealing and higher ICE, whereas fast heating creates extra pores and defects. The heteroatoms (N, S) retained by biomass (such as algae and coffee grounds) can serve as additional Na^+^ adsorption sites.
(3) Closed pores: The shell-based precursor has the highest closed pore volume due to the stability of its lignin network structure. Cellulose-rich materials can be regulated to inhibit graphitization by acid hydrolysis, therefore promoting the formation of closed pores. The thermal stability of starch materials is poor, and pre-carbonization or cross-linking is needed to preserve pores. Although closed pores are very important, a unified understanding of their formation and sodium storage function is still lacking. Recent studies suggest that closed pores form through the transformation of pseudo-graphitic structures during high-temperature carbonization, accompanied by carbon skeleton densification and interlayer spacing shrinkage. Nevertheless, the relationship between the closed pores and the above four precursors still remains unexplored [71].
(4) SEI formation: Residual inorganics (K, Ca, Na salts) in the precursor can act as nucleation sites for local SEI over-growth. Well-developed closed pores with sub-0.35 nm entrances keep electrolytes out, confining SEI to the outer surface—a key factor for long cycle life [72].

To provide a direct comparison, Table 1 summarizes the key electrochemical performance of hard carbon materials derived from the four biomass types. In general, fibrous biomass-derived hard carbons exhibit the best rate performance due to their continuous conductive networks. Granular biomass-derived carbons offer abundant internal pores that contribute to high plateau capacity. Dense biomass-derived carbons provide the highest carbon yield and well-developed closed pores, making them the most promising for industrialization. Special biomass types display high specific capacity with in situ heteroatom doping. These distinct characteristics highlight the importance of precursor selection with a special microstructure for hard carbon anode materials.

## 4. Fabrication Processes and Structural Engineering

Although natural biomass has a structural foundation, it suffers from impurity interference, poor thermal stability, and an uneven structure. A reasonable carbonization process is very important to obtain a high-performance hard carbon anode. The carbonization process usually consists of three steps: preparatory treatment before carbonization, a carbonization process, and post-treatment after carbonization. This section discusses these aspects from four aspects: carbonization temperature, the heating rate and holding time, special carbonization techniques, and post-treatment modification [73].

### 4.1. Preparations Before Carbonization

Appropriate pre-treatment of biomass can significantly improve the quality of the final hard carbon. The pre-treatment involves removing impurities and stabilizing the precursor structure [74]. Biomass naturally contains metal elements such as potassium, calcium, magnesium, and iron. These elements may catalyze local graphitization during carbonization, resulting in uneven layer spacing. At the same time, ash content will occupy active sites, reducing the sodium ion storage capacity. Common methods for removing impurities include acid washing and alkali washing. Acid washing can effectively remove metal ions and increase the layer spacing of hard carbon, therefore increasing the number of active sites and reducing the specific surface area. Wang et al. [75] found that the reversible capacity of hard carbon derived from hazelnut shells after hydrochloric acid reached 342 mAh/g, with 91% of ICE. Alkali washing not only removes impurities but also has the effect of creating pores. Alkali washing usually requires combination with acid washing to achieve the best results [76].

Stabilizing the precursor structure mainly targets heat-unstable raw materials such as starch. Pre-oxidation introduces oxygen-containing functional groups to promote cross-linking and thermal stability [77]. For lignocellulosic biomass (fibrous and dense types), the temperature is kept at 200–280 °C to avoid excessive combustion. For starch-based (granular) precursors, a lower range of 150–250 °C is used to prevent particle melting and agglomeration [78]. Pre-oxidation typically increases carbonization yield by 5–15% compared to direct pyrolysis. The cross-linked network helps retain closed pores, as demonstrated for starch-derived hard carbons [79]. The hard carbon obtained through pre-oxidation has a more abundant closed-pore structure. In addition, pre-carbonization is a low-temperature heat treatment conducted at 200 to 400 °C. Nita et al. [59] shows that pre-carbonization at around 400 °C can effectively remove volatile components, such as water and organic acids. The pre-fixed carbon-rich framework can prevent drastic volume contraction during subsequent high-temperature carbonization. Moreover, hydrothermal treatment can achieve partial hydrolysis and rearrangement of cellulose at a mild temperature range (150 to 250 °C), thereby regulating its crystallinity. The microcrystalline structure and closed-pore formation of the as-prepared hard carbon were optimized.

### 4.2. The Influence of Carbonization Temperature

**Figure 5:**
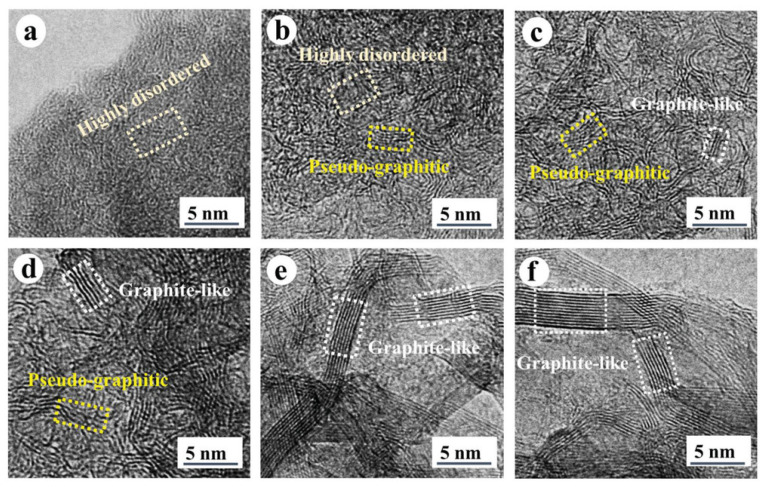
The microstructure of silver leaf charcoal at different pyrolysis temperatures: (**a**) 600 °C; (**b**) 1000 °C; (**c**) 1300 °C; (**d**) 1700 °C; (**e**) 2000 °C; (**f**) 2500 °C. Reprinted with permission from Ref. [84]. Copyright [2019] [Ning Sun].

The carbonization temperature is the most critical factor influencing the microstructure of hard carbon [80]. When the carbonization temperature is below 1000 °C, hard carbon maintains a highly disordered microcrystalline structure. The interlayer spacing at this condition is relatively large, exceeding 0.4 nm. Meanwhile, the material contains a large number of defects and pores [81,82]. The electrochemical characteristic of this structure is that it has a high slope capacity. However, the large specific surface area and numerous defects result in a low ICE. When the carbonization temperature rises to 1000–1600 °C, graphite microcrystals begin to grow. The interlayer spacing gradually shrinks to 0.37–0.40 nm. At the same time, the original pores gradually close, forming a closed-pore structure [83]. The hard carbon prepared during this temperature range has a certain slope capacity, high plateau capacity, and improved ICE. Sun et al. [84] reported that the comprehensive performance of hard carbon is optimal when the carbonization temperature is between 1200 and 1400 °C (Figure 5). When the carbonization temperature exceeds 1600 °C, excessive graphitization results in an interlayer spacing of less than 0.36 nm. Na^+^ cannot be embedded in the hard carbon, corresponding to a low capacity. Therefore, the optimal conditions need to be sought between interlayer spacing, the closed pore rate, and the degree of graphitization [85].

### 4.3. The Influence of Heating Rate and Insulation Time

The heating rate and holding time also have significant effects on the structure of hard carbon. Slow heating is beneficial for defect repair and the slow release of gases. Xiao et al. [8] prepared hard carbon at a heating rate of 0.5 °C/min, achieving 86.1% of ICE. Rapid heating causes rapid gas escape and drastic structural changes, resulting in a large number of pores and defects. In addition, extending the holding time allows graphite microcrystals to grow and arrange fully, therefore enhancing the crystallinity degree and structural uniformity of the material. However, an excessive holding time may lead to excessive crystallization and a decrease in capacity. Moreover, the gradient heating is a precise control method. The typical heat treatment process is as follows: firstly, the raw material is held at 300–400 °C for 1–2 h to completely volatilize low-molecular-weight components; then it is maintained at 600–800 °C for 1 h to facilitate the formation of a stable carbon skeleton; finally, the heating is controlled to reach the target carbonization temperature (1200–1400 °C). This multi-step approach enables the gradual evolution of the structure, yielding a uniform and stable hard carbon structure [86,87].

### 4.4. Special Carbonization Technology

In recent years, some special carbonization techniques have received widespread attention due to their unique functional properties [88]. Catalytic carbonization involves introducing metal salt catalysts (such as iron, nickel, and cobalt) during the carbonization process. The catalysts can reduce the activation energy of the carbonization reaction, enabling them to proceed at lower temperatures. Additionally, the catalysts can promote the ordered growth of graphite microcrystals and the formation of closed pores. Yang et al. [52] improved the plateau capacity and rate performance of hard carbon by introducing catalysts. In addition, the confined carbonization method utilizes templates or the natural pores of biomass to constrain the carbonization process. This method can replicate ideal pore structures. Xu et al. [38] discovered that the natural vascular bundle structure of bamboo can serve as a confinement space, forming continuous ionic transport channels after carbonization. Moreover, transient high-temperature carbonization is an emerging rapid carbonization method, such as Joule heating technology [89]. Qiu et al. [90] reported a flash Joule heating (FJH) strategy capable of carbonizing biomass precursors within seconds. The optimized sample demonstrates an outstanding reversible capacity of 377 mAh/g with a superior ICE of 93.3%. Huang et al. heated biomass precursors to 1950 °C within several seconds via FJH. The contraction of graphite layers was effectively suppressed during the extremely short heating time, resulting in a large layer spacing of approximately 0.4 nm [91,92]. These transient heating techniques offer a promising pathway toward energy-efficient and continuous production of hard carbon materials.

### 4.5. Post-Carbonization Treatment

After carbonization, the basic framework of hard carbon is formed. However, there are a few defects, pores, and residual functional groups on the surface of hard carbon. These factors can affect the electrochemical performance [93,94]. The purpose of post-carbonization treatment is to further optimize these surface issues. Surface coating is one of the most used post-treatment methods. Soft carbon coatings with asphalt-derived carbon and glucose-derived carbon can repair the defects and pores on the surface of hard carbon. The reduction in specific surface area can inhibit electrolyte decomposition and excessive growth of SEI film. Ji et al. [95] improved the ICE of hard carbon from 59.9% to 85.3% with a glucose-derived carbon coating layer. Post-doping with heteroatoms is used to supplement or enhance the deficiencies of natural doping. Heteroatoms, such as nitrogen, phosphorus, sulfur, and boron, can be introduced into biomass through post-treatment. Heteroatom doping can increase defect sites, expand interlayer spacing, and improve electronic conductivity. Gao et al. [48] reported nitrogen and phosphorus dual-doping of starch-based hard carbon. Theoretical calculations indicated that the doping sites have a significantly higher adsorption energy for Na^+^ than that of the intrinsic carbon materials [96]. Control of pore structure is another effective optimization strategy. By blocking the pore entrances through chemical vapor deposition or carbon coating, the conversion from pores to closed pores can be effectively achieved, thereby improving the plateau capacity and ICE [97].

### 4.6. Scalability and Industrial Feasibility

Most reported carbonization protocols are developed at the laboratory scale (grams to hundreds of grams). Translating them to tonne-scale production faces several challenges [98]. In industrial rotary or pusher furnaces, thick particle beds create strong thermal gradients, leading to non-uniform carbonization [99]. The slow heating rates (<1 °C/min) become economically prohibitive. Acid washing generates waste streams. The oxidative stabilization is difficult to apply uniformly to tonne-scale production. Shell-based biomass yields 25–35 wt% carbon, significantly higher than cellulose (15–25 wt%) or starch (10–15 wt%). This directly impacts material cost. Flash Joule heating can carbonize in seconds, offering a potential pathway to continuous, energy-efficient production. Rotary kilns enable better product consistency and heat recovery but require higher capital investment [100,101]. Bridging the gap from laboratory discovery to industrial hard carbon production demands systematic techno-economic assessment and process engineering designed for each biomass type [102].

### 4.7. Chemical and Physical Activation of Biomass-Derived Hard Carbon

Activation is widely employed to enhance the porosity of biomass-derived carbons. While activation dramatically increases specific surface area and pore volume, it also impairs some electrochemical properties of the hard carbon [103,104,105]. Chemical activation typically employs KOH, ZnCl_2_, or H_3_PO_4_. Among these, KOH is the most extensively studied. The activation reaction proceeds as 6KOH + 2C → 2K + 3H_2_ + 2K_2_CO_3_, with subsequent carbonate decomposition generating additional porosity. KOH activation can yield ultrahigh specific surface area values (>2000 m^2^/g). However, several critical trade-offs must be carefully considered. Excessive activation inevitably reduces carbon yield (often to <20 wt%). Introducing abundant oxygen-containing functional groups increases irreversible sodium consumption. Consequently, while high specific surface area enhances slope capacity, ICE decreases markedly. For every 100 m^2^/g increase in specific surface area, ICE decreases by 5–10%. Plateau capacity may be compromised due to insufficient closed pore retention [106]. Physical activation using CO_2_ or steam relies on gasification reactions (C + CO_2_ → 2CO, C + H_2_O → CO + H_2_). Physical activation generally produces less aggressive porosity development, with relatively higher carbon yield and fewer surface functional groups. However, the pore widening effect during prolonged physical activation remains challenging to control.

Excessive activation is generally counterproductive. Recent efforts have explored mild or sequential activation strategies. A moderate specific surface area in the range of 50–300 m^2^/g is often preferred to maintain high ICE while providing sufficient Na^+^ adsorption sites [107]. Systematic studies on the relationship between the activation degree and the electrochemical performance of hard carbon are still lacking, which represents an important direction for future research.

## 5. Conclusions and Perspectives

Biological materials show broad application prospects due to their varied sources, low cost, and chemical stability. Biological-derived hard carbon materials, with inherent structural advantages and chemical properties, have opened new directions for the preparation of anode materials for SIBs. This work systematically reviews the research progress of different structural types of biological hard carbon. The structural characteristics of four biological materials (fibrous, granular, dense, and special) and their influence on the sodium storage performance were summarized. The regulatory effect of carbonization processes on the microstructure of hard carbon was also outlined. These contents provide a reference for the rational design of high-performance hard carbon anode material for SIBs [108].

Several priority areas should guide future work. (1) Precursor standardization with industrial biomass side-streams, such as lignin from biorefineries and potato starch waste, can reduce natural variability [109,110]. (2) Compare pre-oxidation, hydrolysis, and FJH across different precursor families to establish general guidelines. (3) Low-cost heteroatom doping exploits naturally N/S-rich biomass (algae, spent coffee) instead of expensive external reagents. (4) Scalable reactor design: match furnace configuration (fluidized bed, rotary kiln) to the thermal decomposition behavior of each biomass type. (5) Full-cell evaluation: validate hard carbon anodes with realistic cathodes (layered oxides, Prussian blue analogs) regarding energy density, cycle life, and low-temperature performance. (6) Machine learning: use multi-objective optimization to predict the best trade-off among d_002_, defect density and closed pore volume. With these efforts, biomass-derived hard carbon can evolve from a laboratory curiosity to a commercially viable anode material for sodium-ion batteries [111,112].

## Figures and Tables

**Table 1:** Electrochemical performance comparison across four biomass types.

Biomass Type	Precursor	Carbonization Conditions	D_002_ (nm)	Pore Feature	ICE (%)	Reversible Capacity (mAh/g)	Plateau Contribution	Ref.
Fibrous	Bamboo	1300 °C, Ar	~0.382	Microtubules	86.1	326	Moderate	[38]
Fibrous	Cotton	1300 °C	~0.38	Hollow tube	80–85	315	/	[40]
Granular	Potato starch	Pre-oxidation +1400 °C	~0.39	Internal closed pores	~75	280	~100 mAh/g	[48]
Granular	Taro starch	Enzymatic +HT	/	Hierarchical	74.5	278	126 mAh/g	[52]
Dense	Coconut shell	1200–1400 °C	~0.38	Closed pores	80–85	300–350	>50%	[58,59]
Dense	Rice husk	1100–1400 °C	0.38–0.39	Layered	~82	~300	/	
Special	Algae	800–1000 °C	0.37–0.39	N-doped porous	>80	Up to 462	/	[65]
Special	Sugarcane bagasse	1300 °C	/	Fibrous network	75.3	327	~90 mAh/g	[66]

## Data Availability

No new data were created or analyzed in this study. Data sharing is not applicable to this article.
